# Vision-Based Heart and Respiratory Rate Monitoring During Sleep – A Validation Study for the Population at Risk of Sleep Apnea

**DOI:** 10.1109/JTEHM.2019.2946147

**Published:** 2019-10-14

**Authors:** Kaiyin Zhu, Michael Li, Sina Akbarian, Maziar Hafezi, Azadeh Yadollahi, Babak Taati

**Affiliations:** 1 KITEToronto Rehabilitation Institute, University Health Network 7989 Toronto ON M5G 2A2 Canada; 2 Institute of Biomaterial and Biomedical Engineering, University of Toronto 7938 Toronto ON M5S 3G9 Canada; 3 Department of Computer ScienceUniversity of Toronto 7938 Toronto ON M5S 2E4 Canada

**Keywords:** Cardiopulmonary rate, noncontact, sleep disordered breathing, computer vision

## Abstract

A reliable, accessible, and non-intrusive method for tracking respiratory and heart rate is important for improving monitoring and detection of sleep apnea. In this study, an algorithm based on motion analysis of infrared video recordings was validated in 50 adults referred for clinical overnight polysomnography (PSG). The algorithm tracks the displacements of selected feature points on each sleeping participant and extracts respiratory rate using principal component analysis and heart rate using independent component analysis. For respiratory rate estimation (mean ± standard deviation), 89.89 % ± 10.95 % of the overnight estimation was accurate within 1 breath per minute compared to the PSG-derived respiratory rate from the respiratory inductive plethysmography signal, with an average root mean square error (RMSE) of 2.10 ± 1.64 breaths per minute. For heart rate estimation, 77.97 % ± 18.91 % of the overnight estimation was within 5 beats per minute of the heart rate derived from the pulse oximetry signal from PSG, with mean RMSE of 7.47 ± 4.79 beats per minute. No significant difference in estimation of RMSE of either signal was found according to differences in body position, sleep stage, or amount of the body covered by blankets. This vision-based method may prove suitable for overnight, non-contact monitoring of respiratory rate. However, at present, heart rate monitoring is less reliable and will require further work to improve accuracy.

## Graphical abstract

Our proposed algorithm tracks the movement of selected feature points from a video of a sleeping participant, and extracts respiratory rate using principal component analysis and heart rate using independent component analysis.

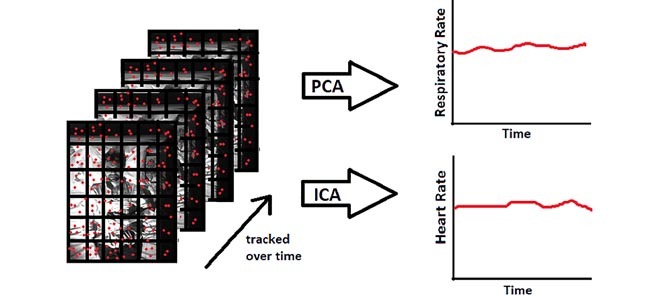

## I. Introduction

Monitoring of respiratory rate, heart rate and respiratory related movement is important for evaluating the quality of sleep and the management of various sleep disorders such as sleep apnea, sleep tachycardia, bradycardia, bruxism, restless leg syndrome and periodic limb movements in sleep. Amongst sleep disorders, sleep apnea is highly prevalent, underdiagnosed, and implicated with various health conditions and excessive daytime sleepiness.

Sleep apnea is defined as the repeated intermittent complete or partial cessation of breathing for 10 seconds or more during sleep, leading to oxygen desaturation and arousals [1]. Sleep apnea disrupts sleep, often resulting in daytime fatigue, and is associated with reduced daily functions and quality of life [2]. Complete cessation of airflow during sleep defines apnea while reduced airflow or thoracoabdominal movement of 30% or more defines hypopnea when associated with ≥ 3 % drop in blood’s oxygen saturation level or arousal [1]. Apneas and hypopneas can be further categorized into central, obstructive, and mixed depending on the underlying mechanism [3].

Between 10–17% of adult men and 3–9% of women are estimated to have obstructive sleep apnea (OSA) [4]. OSA is more common in men, the elderly, and obese population [5]. Numerous studies suggest that individuals with OSA are undiagnosed [6]–[8] Other studies have linked untreated sleep apnea to approximately two fold increase in motor vehicle accidents [9] and occupational accidents [10], as well as increased risk for cardiovascular disease [11], [12], systemic hypertension [13], [14], diabetes [15], and depression [16]. Approximately 50% of patients with heart failure have sleep apnea and 20% of heart-failure related deaths occur at night [17]. The high prevalence of sleep apnea amongst those with cardiovascular complications further compels the need for an accurate, cost-effective, and non-intrusive tracking of heart and respiratory activity during sleep.

Current gold standard for sleep monitoring and assessment is through performing overnight polysomnography (PSG) at a sleep clinic. The process involves attaching over 20 sensors to the sleeper to continuously record electroencephalogram, electrocardiogram, electromyogram, oxygen saturation, nasal flow, and respiratory movement of chest and abdomen walls. Overnight clinical PSG monitoring is expensive [18], wait times are long [19], and can be inconvenient and uncomfortable. Multi-night PSG tests are rarely performed despite large night to night variance in sleep outcomes for those with OSA [20]. Consequently, numerous alternative sleep monitoring technologies have been developed to overcome the disadvantages of full night PSG recording, using reduced number of sensors and allowing for at-home recording [21]. Most consumer technologies come at a cost of reduced accuracy and cannot be used as a stand-alone solution [22], while most clinical modalities still require multiple sensors and a board-certified sleep medicine specialist [23]. Moreover, an attachment of multiple sensors could potentially be inconvenient during sleep. Various contactless technologies were explored for overnight respiratory rate and/or heart rate monitoring including applications of radar [24], WiFi [25], audio recording [26], [27], pressure sensors [28], depth cameras [29], thermal cameras [30], and infrared cameras [31].

Infrared vision based methods are very promising for a few reasons. Firstly, infrared cameras are inexpensive and readily available. Secondly, large amount of additional information can be extracted from visual data, such as sleep position, movements, and surrounding environmental factors. These additional parameters can improve sleep quality assessment and potential diagnosis of other sleep related disorders. A vision-based algorithm using near-infrared video recording for estimating respiratory rate and heart rate was previously developed and validated on healthy awake subjects while simulating sleep condition for short periods of time [31], [32]. Accurately tracked heart rate and respiratory rate can be used in future studies to diagnose sleep apnea. In this paper, we proposed improvements to our previously developed algorithm [31] to address some of the challenges associated with unwanted motion and heart rate estimation during unconstrained nocturnal sleep. We validated the algorithm on sleeping subjects who were referred for a clinical overnight sleep diagnosis.

## II. Materials and Methods

### A. Participants and Sleep Protocol

Subjects between the ages of 18 to 85 were recruited from those who were referred to the sleep laboratory at Toronto Rehabilitation Institute for overnight diagnostic sleep study. The protocol was approved by the ethics boards of University Health Network and the University of Toronto (Research Ethics Board approval number 13-7210-DE). All participants provided written consent before participating in the study. Those with well managed conditions such as hypertension, myasthenia gravis, and diabetes were included. During the study, subjects were not restricted by the number of pillows and blankets used and the clothes worn. No restrictions were placed on the sleep position overnight.

### B. Data Collection

**FIGURE 1.**
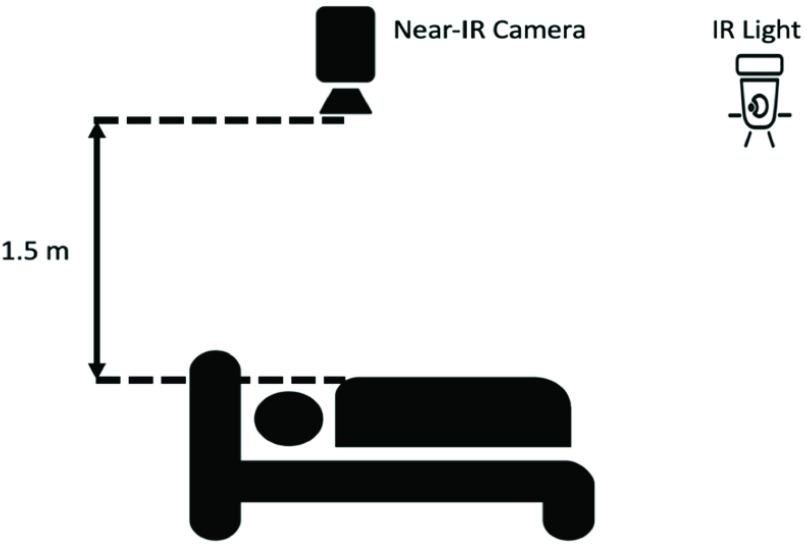
Data collection setup.

**FIGURE 2.**
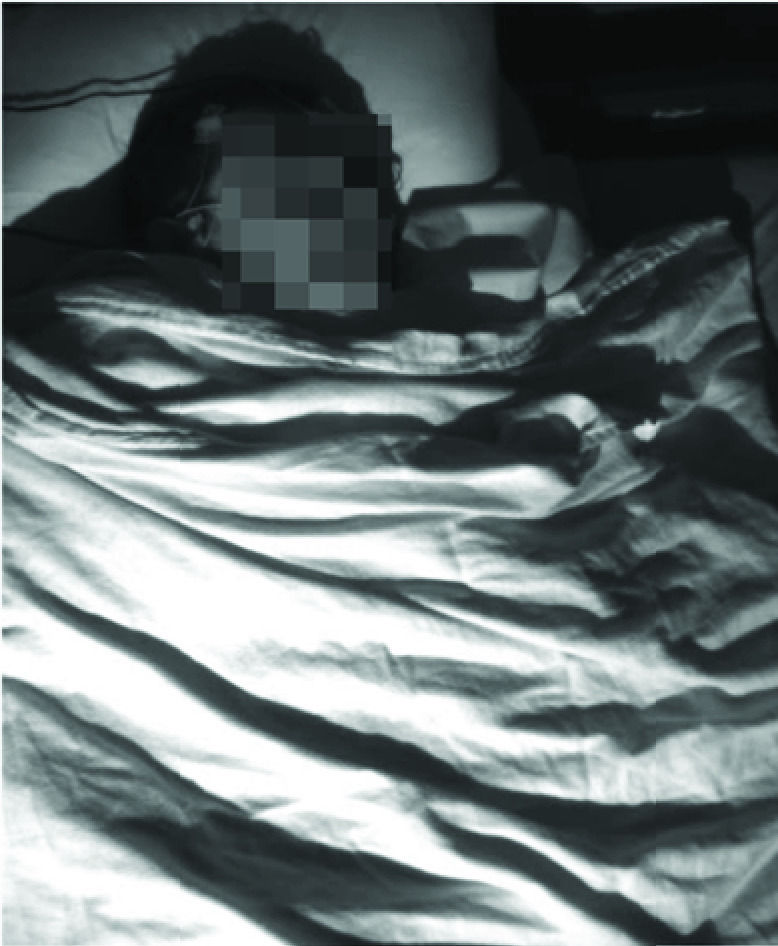
Sample frames.

Videos were captured using Point Grey Firefly MV USB 2.0 monochromatic camera at a resolution of $640 \times 480$ pixels at 30 frames per second. This camera was selected based on its high quantum efficiency in the near infrared range. The camera was mounted approximately 1.5m above the bed to capture the head and torso of the subject. A separate infrared light source (Raytec RM25-F-50), mounted on the ceiling past the foot of the bed, was used for illumination [31]. A schematic of camera set up is shown in Fig. 1 and sample frame (anonymized) that captured by the infrared camera is shown in Fig. 2.

Gold standard assessment of sleep was collected simultaneously using full overnight PSG (Embla s4500). Gold standard heart rate was extracted from one-lead electrocardiogram (ECG) sampled at 250 Hz. The sum of chest and abdominal movement as recorded by respiratory inductance plethysmography (RIP) was sampled at 25 Hz and used to estimate gold standard respiratory rate. Sleep stages, apneas, hypopneas, and any other conditions such as periodic limb movements in sleep, bradycardia and tachycardia were marked by trained sleep technician. Body position, head position, and amount of body covered by sheets were manually annotated by a research analyst from video, while blinded to any respiratory rate and heart rate information.

### C. Data Analysis

Respiratory rate and heart rate estimates are extracted from infrared (IR) video using a computer vision motion-based algorithm previously developed by Li *et al.* [31]. The method tracks the deviation of selected feature points over time. Specifically, the method divides each frame into a grid of 40 pixel by 40-pixel squares. Eight distinct points (corners or textured area) are selected from each grid cell, for a maximum of 1536 points over the whole image. These points are tracked over 30 second windows. To ensure tracked motion is relevant, the maximum frame-to-frame displacement of each tracked point is calculated, and the top and bottom 25 percentile are discarded. The same set of feature points were used for both heart and respiratory rate estimation.

On sliding windows of 30 seconds with 29 seconds overlap, the method applies principal component analysis for respiratory rate estimation and independent component analysis for heart rate estimation. This method compared the frequency spectrum of the output signals from independent component analysis and selected the frequency with highest harmonic periodicity as the heart rate estimation [31]. The harmonic periodicity is determined by taking the sum of the amplitudes of the fundamental frequency and its first two harmonics divided by the total spectral density. However, this method can consider respiratory harmonics as the estimation for heart rate. To overcome this challenge, we scored the top candidate frequencies, up to 12 candidates, based on its harmonic periodicity, proximity to heart rate estimation in the previous window, and likelihood of the frequency as a heart rate candidate. The score $S$ was calculated for each candidate frequency by the following equation:

\begin{equation*} S = 2
              s_{1}+(1-s_{2})+{(1-s}_{3})\tag{1}\end{equation*}

$s_{1}$ is the harmonic periodicity of candidate frequency divided by the sum of harmonic periodicity over all candidate frequencies, $s_{2}$ is the normalized absolute difference between previous heart rate estimate and current candidate frequency, and $s_{3}$ is the normalized absolute difference between a reasonable heart rate and current candidate. For this study, reasonable heart rate was set to 80 beats per minute (bpm), which is the midpoint of the 20 bpm to 140 bpm range considered for heart rate estimation. The candidate with highest score was selected as the heart rate estimate.

The gold standard heart rate and respiratory rate were calculated from ECG and RIP signals respectively. Instantaneous heart rate was calculated by detecting R-peaks from ECG signal using parabolic fitting, and then averaging 5 consecutive R-R intervals. The instantaneous heart rate was averaged over a 30-second moving window with 29 second overlap as the gold standard heart rate. The gold standard respiratory rate was extracted from the same moving window using the same method as Li *et al.* [31].

One of the main challenges of the vision based method [31] is accurate prediction of heart rate and respiratory rate during movements such as position change and limb movement, during which large artefacts are also seen in the gold standard measurements. In this study, the algorithm was modified such that when large spikes in displacement of the tracked feature points was detected, new reference set of feature points was sampled. Also, the 30 seconds windows including and following the large motion were labelled as ‘moving’ and excluded from analysis. Segments, in which the participant was not seen in the frame, either due to the subject moving out of bed or over exposure of the video frame, were excluded from analysis. In addition, segments where gold standard heart rate was outside the range of 20–140 bpm and gold standard respiratory rate was outside the range of 1–100 breaths per minute (br/min) were also excluded. The maximum values of the considered range were intentionally set higher than realistic values during sleep for robustness.

### D. Estimation Comparison

For each subject, percent of prediction within 1 br/min or 5 bpm of gold standard, percent of prediction within 0.5 br/min or 2.5 bpm of gold standard, root mean square error (RMSE), and mean absolute error (MAE) between estimated and gold standard respiratory rate and heart rate were calculated. Kruskal-Wallis statistical test was performed with multiple comparison test to determine whether significant difference in predictive performance can be found between various sleep-awake stages, sleep positions, amount of body covered, respiratory events, and periodic limb movement severity. P-value < 0.05 was considered significant. Pearson’s correlation between estimation error measures and age, BMI, AHI, obstructive AHI, central AHI, arousal index, movement, periodic limb movement index, number of position changes, REM sleep percentage, sleep efficiency, or variance of gold standard respiratory rate or heart rate were calculated.

The proposed method of respiratory rate and heart rate were compared against other available non-contact methods for estimating respiratory rate [33] and heart rate [34]. To determine the importance of estimating respiratory rate and heart rate variability overnight, the proposed method was also compared against mean hold method where average gold standard measurement from the first 10 minutes of recordings were used as the respiratory and heart rate estimations for the rest of the night.

## III. Results

53 subjects were recruited for this study. Three subjects were excluded from analysis due to missing video data (connection failures), leaving total of 50 subjects for analysis. 30 male and 20 female subjects were included with average age of 53 ± 15 years old and BMI of 29.4 ± 6.4 kg/$\text{m}^{2}$. Participant characteristics are stratified by Apnea Hypopnea Index (AHI) and summarized in Table 1. AHI of less than 5 is defined as no sleep apnea, 5 to 15 as mild, 15 to 30 as moderate, and greater than or equal to 30 as severe sleep apnea. 12 subjects were not apneic, 13 were mild, 11 were moderate and 14 were severe. 48 subjects had obstructive hypopneas, 34 subjects had obstructive apneas, 19 subjects had central hypopneas, 22 subjects had central apneas, and only 3 subjects had mixed apneas.

**TABLE 1:** Subject Characteristics

Characteristics	AHI < 5	5 ≤ AHI < 15	15 ≤ AHI < 30	AHI ≥ 30	All
Number (Male)	12 (4)	13 (9)	11 (7)	14 (10)	50 (30)
Age, year	45.5 ± 16.0	55.0 ± 14.1	50.1 ± 9.1	59.7 ± 16.1	53.0 ± 14.9
Body mass index, kg/m^2^	28.5 ± 5.8	26.7 ± 4.1	28.0 ± 4.8	33.6 ± 8.1	29.4 ± 6.4
AHI, events per hour	3.1 ± 1.4	8.9 ± 2.7	22.3 ± 4.0	60.9 ± 34.3	25.4 ± 29.5
Obstructive AHI, events per hour	2.7 ± 1.7	7.6 ± 3.7	19.1 ± 7.9	55.6 ± 38.5	22.4 ± 29.7
Central AHI, events per hour	0.4 ± 0.9	2.6 ± 7.1	3.1 ± 6.0	3.8 ± 10.1	2.5 ± 7.0
Total sleep time, hour	5.4 ± 1.2	5.2 ± 1.4	5.3 ± 1.3	4.8 ± 1.6	5.2 ± 1.4
Sleep efficiency, %	78.2 ± 12.5	77.8 ± 15.6	76.7 ± 13.5	70.0 ± 24.0	75.5 ± 17.2
REM sleep percentage	13.6 ± 5.6	16.0 ± 7.2	17.8 ± 7.0	12.9 ± 8.5	15.0 ± 7.3
Arousal index, arousals per hour	18.1 ± 10.2	17.1 ± 7.7	24.9 ± 10.5	54.9 ± 33.0	29.6 ± 24.7
Count of sleep position changes	76 ± 21	77 ± 24	89 ± 22	110 ± 56	89 ± 37
Average magnitude of movement, arbitrary units	27.8 ± 16.4	32.7 ± 22.2	37.9 ± 22.1	28.1 ± 18.3	31.4 ± 19.7
Periodic limb movement index, movements per hour	23.7 ± 37.1	11.4 ± 16.3	11.6 ± 18.6	24.1 ± 34.6	17.9 ± 28.3
Average respiratory rate, breaths per minute	15.8 ± 3.1	15.2 ± 3.1	14.7 ± 1.1	17.4 ± 3.6	15.9 ± 3.0
Variance of respiratory rate, breaths per minute squared	3.1 ± 2.4	3.1 ± 2.4	2.4 ± 1.3	7.4 ± 9.1	4.1 ± 5.4
Average heart rate, beats per minute	68.4 ± 10.5	68.3 ± 8.5	63.0 ± 8.2	76.1 ± 10.7	69.3 ± 10.5
Variance of heart rate, beats per minute squared	21.6 ± 16.7	14.9 ± 8.7	12.2 ± 8.9	17.3 ± 12.5	16.6 ± 12.3

Characteristics of subjects collected from overnight polysomnography and sleep technician annotations. Data is presented in mean ± standard deviation. AHI = apnea hypopnea index, REM = rapid eye movement.

The average duration of sleep with normal breathing among all subjects was 215.2 ± 99.2 minutes. The average durations of respiratory event segments among all subjects were 50.4 ± 53.7 min for obstructive hypopneas, 11.2 ± 20.0 min for obstructive apneas, 6.4 ± 8.3 min for central hypopneas, 3.1 ± 5.0 min for central apneas, and 14.0 ± 21.4 min for mixed apneas.

**FIGURE 3.**
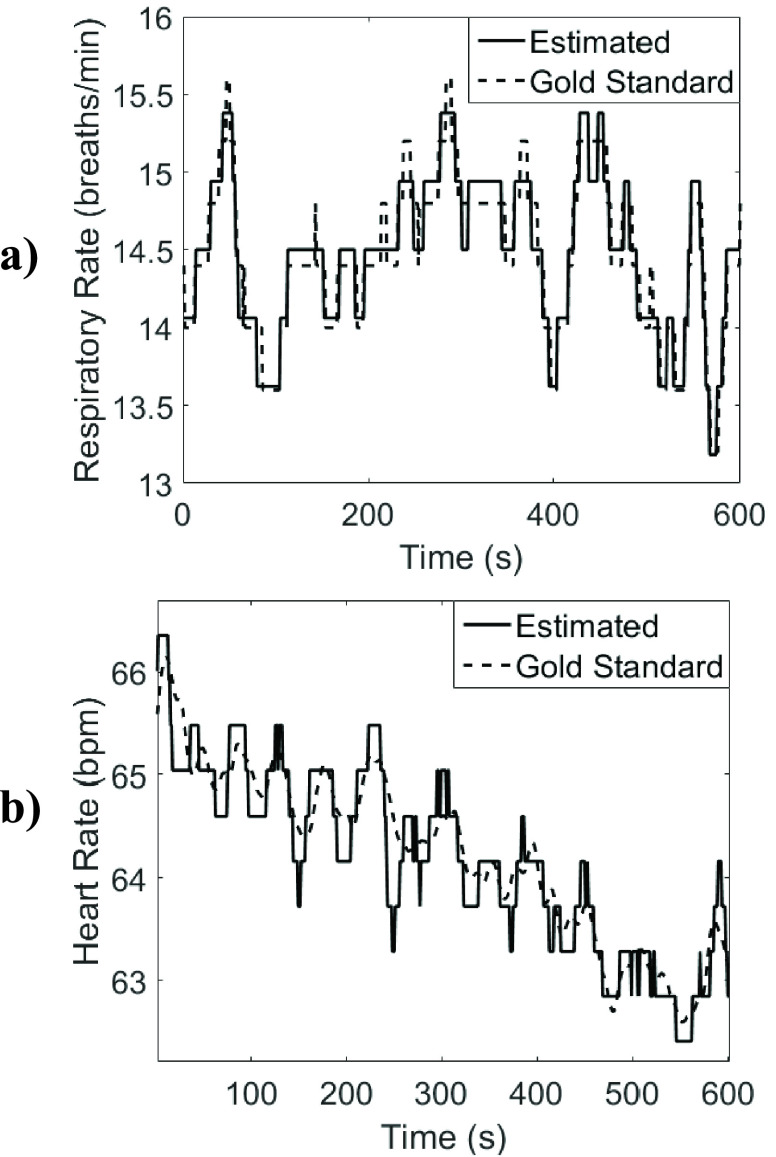
Example segment of estimated a) respiratory rate, and b) heart rate compared to gold standard rates from the sum of respiratory inductance plethysmography signal and from electrocardiogram signal respectively. (Subject 16, during uncovered right lateral NREM sleep).

All subjects except four had full sleep cycle including NREM and REM sleep stages. Out of the 50 subjects included for respiratory rate analysis, 5 were excluded from heart rate analysis due to frequent irregular heart activities, such as premature ventricular contractions, atrial fibrillations, or atrioventricular block type II, as indicated by the sleep technician based on the PSG. The average percent of recorded duration excluded from analysis over all participants was 15.46 ± 13.05%; 12.83 ± 9.79% of which were segments with movement, 1.03 ± 1.78% were segments with error in gold standard measurements or participant was not visible in the frame of the video, and 1.61 ± 3.81% were when the algorithm did not detect any feature point. An example of the gold standard and predicted heart rate and respiratory rate are shown in Fig. 3.

Table 2 summarizes the results of respiratory rate and heart rate estimation during wakefulness, NREM or REM sleep with normal breathing, and during segments with apneas or hypopneas. For respiratory rate estimation, 89.89 ± 10.95 % of the estimated values were within 1 br/min of the gold standard, 83.65 ± 13.91 % were within 0.5 br/min, average RMSE was 2.10 ± 1.64 br/min, and average MAE was 0.82 ± 0.89 br/min. For heart rate estimation, 77.97 ± 18.91 % of the estimated segments were within 5 bpm of the gold standard, 67.60 ± 20.73 % were within 2.5 bpm, average RMSE was 7.47 ± 4.79 bpm, and average MAE was 4.36 ± 3.69 bpm. RMSE for estimating respiratory rates were significantly different between awake segments and sleep segments with normal breathing (p < 0.0001) and between sleep segments with normal breathing and respiratory events (p < 0.0001). RMSE for estimating respiratory rates were similar between NREM and REM sleep and between awake and respiratory event segments. No significant difference in median heart rate estimation RMSE was found for any pairwise comparison between awake, REM sleep with normal breathing, NREM sleep with normal breathing, and sleep with respiratory events.

**TABLE 2:** Respiratory and Heart Rate Estimation Error

Respiratory Rate Estimation mean (± std)	E < 1 br/min (%)	E < 0.5 br/min (%)	RMSE (br/min)	MAE
Awake, N = 50	83.32 (± 13.85)	75.36 (± 16.34)	2.85 (± 1.82)	1.24 (± 1.13)
NREM, N = 50	94.63 (± 9.16)	90.03 (± 12.30)	1.29 (± 1.26)	0.49 (± 0.66)
REM, N = 46	92.72 (± 11.61)	86.79 (± 14.33)	1.34 (± 1.44)	0.55 (± 0.63)
Apnea and Hypopnea, N = 50	83.04 (± 13.46)	73.40 (± 17.28)	2.74 (± 1.89)	1.23 (± 1.07)
All, N =50	89.89 (± 10.95)	83.65 (± 13.91)	2.10 9 (± 1.64)	0.82 (± 0.89)
Heart Rate Estimation mean (± std)	E < 5 bpm (%)	E < 2.5 bpm (%)	RMSE (bpm)	MAE
Awake, N = 45	75.42 (± 19.20)	64.60 (± 21.00)	8.57 (± 5.51)	5.13 (± 4.34)
NREM, N = 45	80.18 (± 20.81)	71.06 (± 22.68)	6.81 (± 5.34)	4.05 (± 4.41)
REM, N = 42	78.66 (± 22.79)	68.69 (± 23.95)	6.93 (± 5.16)	4.26 (± 4.05)
Apnea and Hypopnea, N = 45	75.34 (± 20.78)	60.05 (± 22.92)	7.47 (± 5.41)	4.90 (± 4.31)
All, N = 45	77.97 (± 18.91)	67.60 (± 20.73)	7.47 (± 4.79)	4.36 (± 3.69)

Error values were calculated by averaging over all subjects. std = standard deviation, br/min = breaths per minute, bpm = beats per minute, E = percent of estimation for which the absolute error is less than specified threshold, RMSE = root mean squared error, MAE = mean absolute error, N = number of subjects, NREM = non-rapid eye movement sleep, REM = rapid eye movement sleep.

**FIGURE 4.**
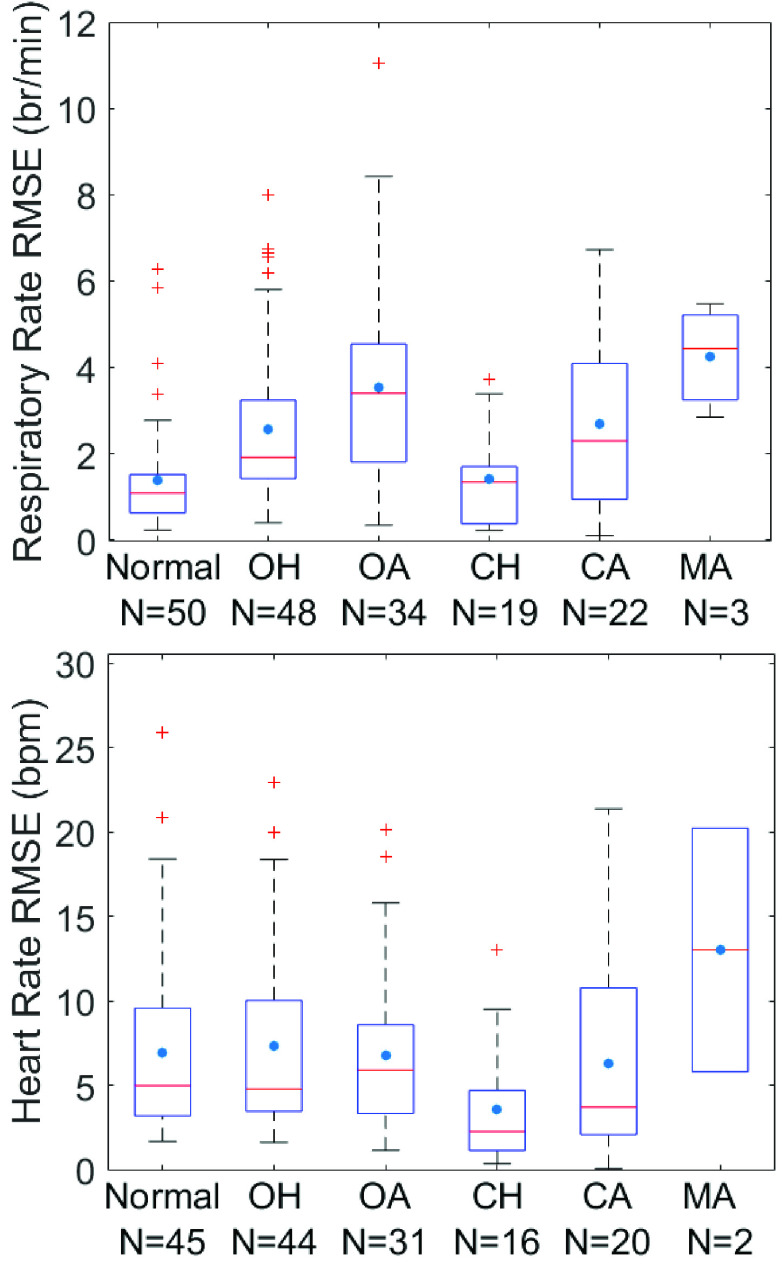
Quartile boxplot of root mean square error(RMSE) by subject of a) respiratory rate and b) heart rate, for normal breathing sleep segments, obstructive hypopneas(OH), obstructive apneas (OA), central hypopneas(CH), central apneas(CA), and mixed apneas(MA).

Fig. 4 shows the RMSE values for respiratory rate and heart rate estimation among different subjects during normal breathing and various respiratory events during sleep. Median (interquartile range) RMSE for respiratory rate estimation during normal breathing segments was 1.09 (0.63–1.48) br/min; similar during central hypopnea at 1.34 (0.38–1.70) br/min (p > 0.1); higher during obstructive hypopnea segments at 1.92 (1.42–3.17) br/min (p = 0.003), obstructive apnea segments at 3.39 (1.82–4.52) br/min (p < 0.0001), and central apneas at 2.30 (0.96–4.06) br/min (p = 0.036). Median heart rate estimation RMSE across subjects was 4.99 (3.27–9.52) bpm during normal breathing segments, 4.79 (3.48–9.92) bpm and 5.92 (3.35–8.58) bpm for obstructive hypopneas and apneas respectively, 2.27 (1.30–4.41) bpm and 3.72 (2.09–10.77) bpm for central hypopneas and apneas respectively, and 13.02 (9.42–16.62) bpm for mixed apneas. The median heart rate estimation RMSE was lower during central hypopneas compared to sleep segments with normal breathing, obstructive hypopneas and obstructive apneas (p < 0.01 for all).

**FIGURE 5.**
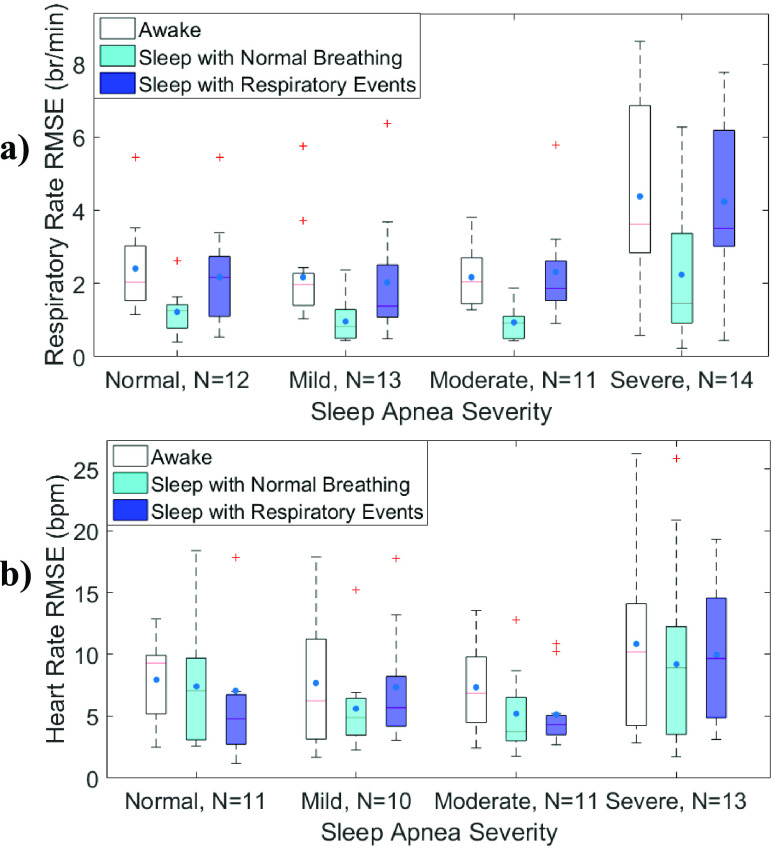
Quartile boxplot of root mean square error of a) respiratory rate and b) pulse for or non-apneic (AHI < 5), mild (5 ≤ AHI < 15), moderate (15 ≤ AHI < 30), and severe (AHI ≥ 30) sleep apnea subjects during awake, sleep with normal breathing, or sleep with apneas or hypopneas.

Fig. 5 shows details of RMSE during awake, sleep with normal breathing and respiratory events for participants with different severities of sleep apnea. For all sleep apnea severities, RMSE of respiratory rate estimation was significantly lower during sleep segments with normal breathing than awake segments (p < 0.02 for all) and sleep segments with respiratory event (p < 0.02, for all). While awake, the RMSE for respiratory rate estimation was significantly higher for subjects with severe sleep apnea compared to those with no, mild and moderate sleep apnea (p ≤ 0.007 for all). Similarly, during respiratory events, the RMSE for respiratory rate estimation was significantly higher for subjects with severe sleep apnea compared to those with mild or moderate sleep apnea (p ≤ 0.006 for both). RMSE of heart rate estimation was similar among subjects with different severities of sleep apnea, for awake segments, and sleep segments without and with respiratory events (p ≥ 0.3 for all).

**FIGURE 6.**
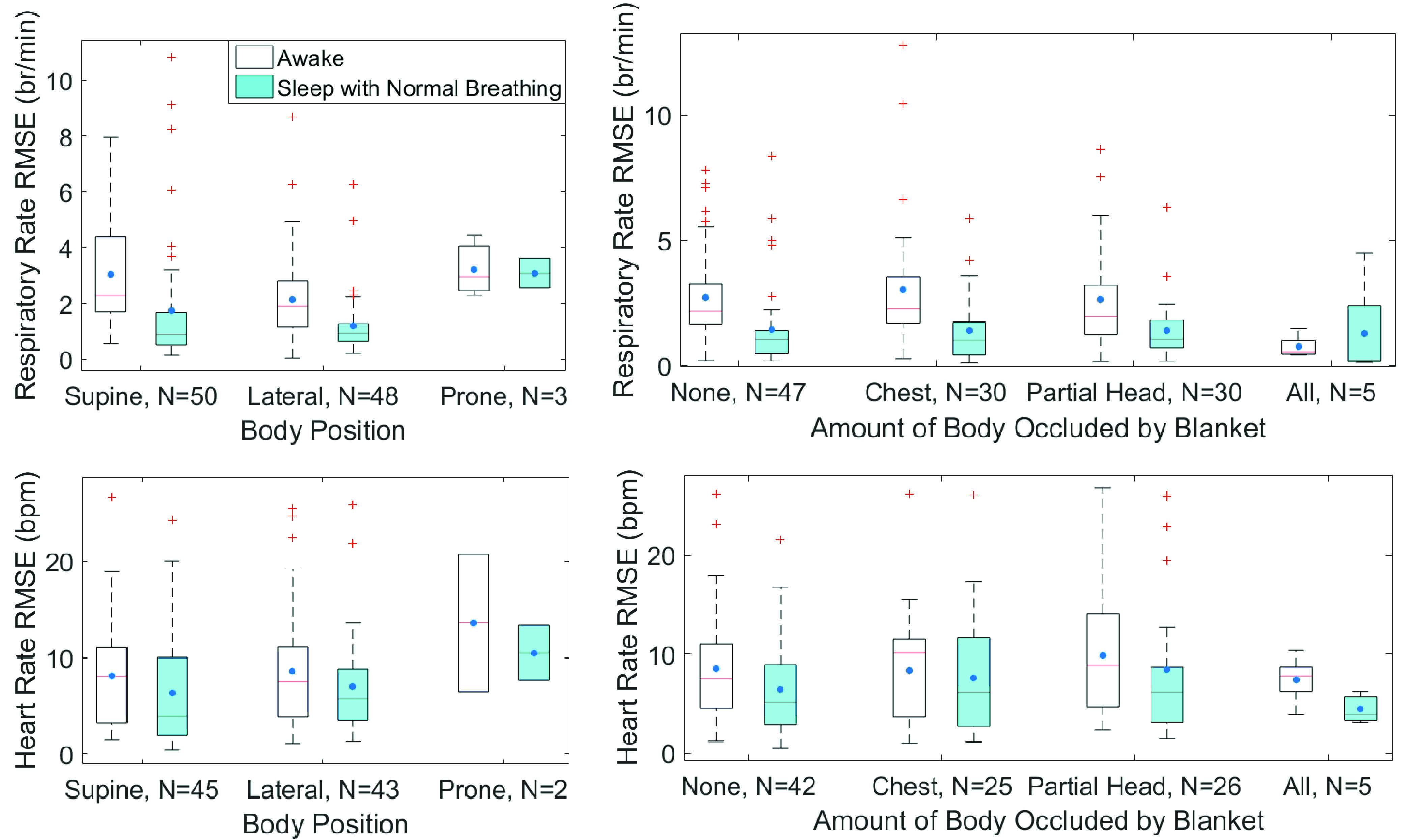
Quartile boxplot of root mean square error of respiratory rate (top) and heart rate (bottom) for each subject separated by body position (left) and amount of body occluded by blanket (right) during awake or sleep with normal breathing.

Out of the 50 subjects, all had spent time in supine posture, 48 had also slept in lateral side, and 3 had data in prone position. The RMSE for respiratory rate estimation was statistically higher while awake compared to supine and lateral positions (Fig. 6a, p ≤ 0.0001) and for various amount of blanket occlusion (Fig. 6b, p ≤ 0.0010) except for the case of completely covered. While awake, RMSE for respiratory rate estimation was significantly lower in lateral position compared to supine (p = 0.03). RMSE for heart rate estimation was similar for various sleep positions and the amount of body covered. There was no significant difference in RMSE for respiratory rate and heart rate estimation for varying severities of periodic limb movement.

For respiratory rate estimation, RMSE was strongly correlated with variance of gold standard respiratory rate (r = 0.80, p < 0.0001). For heart rate estimation, RMSE was correlated with obstructive hypopnea and apnea index (r = 0.65, p < 0.0001).

## IV. Discussion

The most important finding of this study is to modify and validate a vision-based non-contact monitoring system to estimate respiratory rate and heart rate during sleep. We achieved promising results for estimating respiratory rate in various sleeping positions and amount of blanket coverage. While the error of respiratory rate estimation was generally higher during awake periods and respiratory events during sleep, it was still less than 2.8br/min. This was expected as there are more non-periodic movements during wakefulness and obstructive apneas and hypopneas. For respiratory rate estimation, in 43 out of the 50 subjects, the error was less than 1 br/min more than 85% of the night. For heart rate estimation, in 32 out of the 45 subjects, error was less than 5 bpm more than 70% of the night. Importantly, Our method for respiratory and heart rate estimation is unaffected by the sleep position, amount of the body covered by blanket, and PLM severity during natural sleep with normal breathing.

Our proposed method for respiratory rate estimation is more accurate during normal breathing while asleep compared to wakefulness and respiratory events during sleep. There are a few factors that may have contributed to the reduced predictive accuracy and precision of respiratory rate estimation during sleep with respiratory events, and especially for subjects with severe apnea. Subjects with severe sleep apnea have frequent arousals and associated increases in head movement. The respiratory patterns of subjects with severe sleep apnea are also highly variable during sleep including: hyperventilation after an event, paradoxical breathing during obstructive events, and reduced ventilatory effort during central events. The highly variable respiratory patterns and movements during obstructive events can reduce the accuracy of our proposed method which relies on periodicity of the signal. Nonetheless, our proposed method for respiratory rate estimation outperforms the method proposed by Nakajima *et al.* [33] and mean-hold as seen in Table 3. Future studies could include larger sample size to increase robustness of the proposed algorithm for different breathing patterns during sleep and for various severities of sleep apnea.

**TABLE 3:** Respiratory and Heart Rate Estimation Performance Comparison

Respiratory Rate Estimation E < 1 br/min (%) mean (± std)	Li$et~al$.	Nakajima $et~al$. [33]	Mean Hold
Awake, N = 50	83.32 (± 13.85)	56.63 (± 17.78)	28.72 (± 14.42)
Normal sleep, N = 50	94.20 (± 8.88)	74.11 (± 19.42)	32.46 (± 22.33)
Apnea and hypopnea, N = 50	83.04 (± 13.46)	60.36 (± 20.51)	26.12 (± 15.64)
All, N = 50	89.89 (± 10.95)	69.10 (± 18.77)	31.75 (± 17.37)
Processing time per predicted second	0.875s (± 0.116s)^*^	2.824s (± 0.229s)	N/A
Heart Rate Estimation E < 5 bpm (%) mean (± std)	Li$et~al$.	Balakrishnan $et~al$. [34]	Mean Hold
Awake, N = 45	75.42 (± 19.20)	38.60 (± 21.82)	66.83 (± 20.94)
Normal sleep, N = 45	79.91 (± 20.67)	49.91 (± 26.34)	58.43 (± 31.49)
Apnea and hypopnea, N = 45	75.34 (± 20.78)	46.97 (± 24.03)	54.28 (± 30.)
All, N = 45	77.97 (± 18.91)	47.77 (± 24.09)	58.47 (± 28.17)
Processing time per predicted second	0.875s (± 0.116s)^*^	0.656s (± 0.090s)	N/A

Performance measure compared is percentage of recorded duration for which respiratory and heart rate estimates are within 1 breath/min and 5 beats per minute respectively. Processing time was calculated by taking the average run times for the analysis of the first 15 minute segment for each subject. Values shown are the mean over all subjects.

std = standard deviation, br/min = breaths per minute, bpm = beats per minute, E = percent of estimation for which the absolute error is less than specified threshold, N = number of subjects,

^*^ Combined processing time for respiratory rate and pulse estimation

Estimating heart rate based on motion tracking is more challenging than respiratory rate estimation. Our proposed method for heart rate estimation achieved reasonable accuracy for normal breathing during sleep. One source of error in heart rate estimation is the residual respiratory motion harmonics. This is supported by the fact that RMSE for heart rate estimation was lower during central respiratory events, for which respiratory effort is minimum or zero. Overall, our proposed method for heart rate estimation outperformed the methods proposed by Balakrishnan *et al*. [34] and mean-hold when comparing percentage of estimation within 2.5bpm or 5bpm of gold standard as seen in Table 3. Future work could consider setting a more adaptive bound on the expected values of heart rate to improve heart rate estimation accuracy.

One limitation of estimating respiratory rate and heart rate through this vision based motion tracking was the exclusion of segments with large body movements. Our method was unable to isolate respiratory and heart beat during periods with large movements. As well, a new set of feature points needed to be selected and tracked after each body position change, which resulted in a minimum of 30 second segments with no respiratory rate and heart rate estimation on each occurrence. Participants with irregular cardiac activities were also excluded from heart rate estimation. Only 3 participants slept in the prone position for this study; the sample size would need to be increased significantly to determine the performance of our algorithm during sleep in prone position. Another limitation of this study pertains to the thin sheets and blankets used by the participants. Thicker blankets and airier covers could reduce the amount of motion captured by the camera from a distance, which could lead to reduced predictive accuracy in realistic home environments. Another limitation of this study is the limited number of baseline methods used for comparisons. Baselines used (Nakajima *et al.* [33] and Balakrishnan *et al*. [34]) were chosen based on the availability of code and also based on the ability of these approaches to process monochromatic video by analyzing motion.

## V. Conclusion

Through tracking the motion of upper torso and head, algorithms developed by our group (Li et al., 2017) were experimentally validated and shown to be able to estimate respiratory rate during sleep with high accuracy. In the future, a non-contact sleep monitoring system can be developed for the home environment based on our algorithm and tested against available portable sleep monitoring devices. A non-contact solution is crucial for enabling long-term continuous monitoring and studying of various nocturnal conditions, such as monitoring for sudden infant death syndrome, nocturnal asthma, or nocturnal seizures. For diagnosis of sleep apnea, changes in respiratory volumes may be estimated through tracking changes in the magnitude of chest and abdominal movements with high accuracy. Further differentiation for central versus obstructive sleep apnea, or for positional OSA, can also be possible through motion analysis. This would significantly improve the utility of at-home sleep monitors.
